# A Miniaturized Two-Electrode Detection System for Convenient and Rapid Ferricyanide-Mediated Chemical Toxicity Screening

**DOI:** 10.1021/acs.analchem.6c00076

**Published:** 2026-03-30

**Authors:** Krittamate Buppasirakul, Wipa Suginta, Albert Schulte

**Affiliations:** School of Biomolecular Science and Engineering, Vidyasirimedhi Institute of Science and Technology (VISTEC), Wang Chan Valley, Rayong 21210, Thailand

## Abstract

Toxicity bioassays play a crucial role in chemical risk assessment; however, conventional methods usually require specialized equipment, and limited accessibility is a significant drawback. Here, we present a user-friendly version using a simple two-electrode ferricyanide-mediated scaling of microbial respiration for rapid, convenient, and reliable toxicity assessment. The electrochemical cell is designed for continuous pulse-amperometric analysis, eliminating the need for disassembly/assembly and enabling a straightforward protocol to obtain a comprehensive evaluation of bacterial viability across a specific toxicant gradient in a single test run. Dose–response toxicity trends were directly observable, and half-maximal inhibitory concentration (IC_50_) values were derived for comparative analysis. For a representative set of toxic chemicals, our assay produced an IC_50_ ranking of Ni^2+^ > Cd^2+^ > Pb^2+^ for heavy metal ions and DCP > PNP > carbaryl for organic toxicants. The IC_50_ values correlate well, in most cases, with results from other electrochemical toxicity assays and Microtox data. Our specialized bioassay enables a simple, accessible, cost-effective, and rapid toxicity assessment, providing instant toxicity clarification and making it suitable as a first-line screening tool. Additionally, the method can potentially be included in a broader toxicity assessment battery and has feasible applicability in other fields.

## Graphical abstract

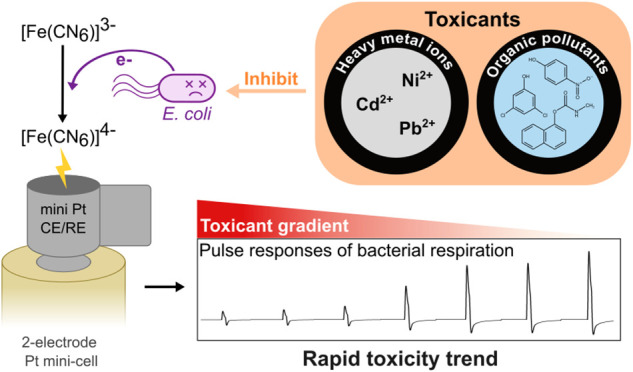

## Introduction

Before being brought to market and widespread use is permitted, chemicals are inspected for their impact on the environment and human, animal, and plant health. The assays required for toxicity testing are not only crucial for accurate predictions of possible toxic traits, but their data is also the foundation of safe chemical application and protective threat mitigation.^1–5^ Toxicity bioassays on suitable model microorganisms assess the cellular vitality risk, enabling straightforward interpretation without, for instance, the ethical concerns of animal experiments, while providing the benefits of rapid results and adequate effectiveness.^6–10^ Microtox is an example of a widely accepted toxicity bioassay format, often serving as the gold standard, that measures the light emission by luminescent bacteria that survived exposure to test toxicants.^11–13^ Microtox’s proven capability to evaluate the hazardous content of environmental, food, and pharmaceutical samples highlights its suitability for reliable toxicity testing.^14–17^ However, two major drawbacks of this gold-standard approach are the involvement of expensive, specific optical equipment and the restriction of working with luminous bacteria. These factors reduce its general accessibility, particularly in resource-constrained environments, and restrict the range of microbial options available for use.

Microbial toxicity bioassays with an electrochemical instead of an optical luminescence readout have been used recently as a promising option, complementary to Microtox. Dissolved redox mediators that contact enzymes on the periplasmic side of the inner lipid membrane, shuttling electrons from the cell’s respiratory chain to suitable working electrodes in the test solution,^18,19^ are effective functional assay components that enable proportional viability analysis in the presence of toxicants.^20^ A clear practical advantage of this method is that the cellular viability testing can be carried out with a wide range of microorganisms, including bacteria,^21–24^ yeasts,^25,26^ and even microbial consortia.^27,28^ Versatility in microbe compatibility is obviously accompanied by the opportunity to tailor the properties of the engaged redox mediator, which can be lipophilic,^29,30^ hydrophilic,^21,22,24,31^ or mixed.^25,26,28^ Other assets of the methodology are the high sensitivity, rapidity, and simplicity of the electrochemical measurement protocols.^20^ Furthermore, the workhorses for electrochemical toxicity testing, the potentiostats, are readily available at low cost from many global suppliers. If used in miniaturized space-saving designs such as USB stick-sized units with mobile phone or cloud connectivity or in a compact hand-held format, portability is ensured, and on-site applications are practical and convenient.

Logically, the speed of toxicity assay data acquisition matters, but the time needed for subsequent data analysis and final toxicity interpretation is equally important. Most toxicity assays estimate the risk of chemicals by classifying them according to their half-maximal inhibitory concentration (IC_50_) values, which are extracted from dose–response curves constructed by using actual trial data. When, however, a microbe of choice is exposed in a toxicity test to a gradient of toxicant, an assay may also quickly reveal toxicity trends, the lowest inhibitory concentration, and provide a simple “toxic/safe” output, a rapid qualitative indication of microbial vitality or toxicity strength. A common illustration of this toxicity-evaluation strategy is the rapid, visible color shift that occurs in dye-based microbial-vitality assays with changes in the toxicant concentration, making risk trends instantly observable.^32–34^

The strategy of assaying toxicity as a function of toxicant concentration can be applied to analytical platforms other than optical ones. Previously, we introduced an electrochemical method for scanning bacterial viability across antibiotic concentration ranges, allowing convenient amperometric profiling of *Escherichia coli* antimicrobial susceptibility and easy assessments of the lowest inhibitory concentration for *E. coli* growth.^44,45^ In this work, we extended this approach to ferricyanide-mediated electrochemical toxicity assessments of *E. coli* exposed to heavy metal ions and organic pollutants. The core of the proposed chemical toxicity test is a simple, miniaturized two-electrode cylinder that allows consecutive analysis of ferrocyanide production by bacterial respiration at different toxicant concentrations, eliminating the need for exhaustive and repetitive cell disassembly and reassembly, as is required in beaker cells. The pulse-amperometric signals obtained show dose–response characteristics directly at the time of the trial run, facilitating quick interpretation of toxicants’ potency within a range of concentrations. These dose–response pulses also enable the evaluation of IC_50_s for the tested compounds and show good agreement with results from other electrochemical studies and Microtox assessments. The proposed technique is an accessible complementary tool for rapid chemical toxicity testing and is ideal for effective preliminary screening of a chemical safety risk.

## Experimental Section

### Materials

All reagents were of analytical grade. Nickel­(II) acetate tetrahydrate, cadmium acetate hydrate, lead­(II) acetate trihydrate, 4-nitrophenol, and carbaryl were purchased from Sigma-Aldrich. 3,5-Dichlorophenol was obtained from the Tokyo Chemical Industry. Potassium ferricyanide was acquired from Thermo Fischer Scientific. Ammonium formate was purchased from Acros Organics. Ultrapure water was utilized throughout the experiments.

Toxicant stock solutions were prepared at a concentration of 640.0 mg/L using the appropriate solvents: Ni^2+^, Cd^2+^, and Pb^2+^ in 2% HNO_3_, 3,5-dichlorophenol (DCP) in ethanol, 4-nitrophenol (PNP) in water, and carbaryl in DMSO. Sterilization of the stock solutions was achieved using 0.22-μm syringe filters.

### Bacterial Cultivation

The Gram-negative model bacterium *E. coli* DH5α (Invitrogen) was retrieved from a glycerol stock stored at −80 °C and cultivated overnight on agar plates to yield isolated bacterial colonies. A single colony was then cultivated in sterile liquid Luria–Bertani (LB) broth containing 10 g/L tryptone, 10 g/L NaCl, and 5 g/L yeast extract at a pH of 7.0 ± 0.2, at 37 °C, with continuous shaking at 180 rpm for 16 h. The resulting stationary-phase inoculum was adjusted to the desired optical density at a wavelength of 600 nm (OD) to establish the working inoculum, with standard plate counting indicating that OD = 1.0 = 1.5 × 10^8^ CFU/mL.

### Toxicant Incubation

The model toxicants in this work are heavy metal ions (Ni^2+^, Cd^2+^, and Pb^2+^) and organic pollutants (DCP, PNP, and carbaryl). Toxicant solutions were diluted 2-fold from the stocks to yield sample solutions ranging from 0.25 to 128 mg/L, except for Pb^2+^, for which a multiplier of 4 was used, ranging from 12 to 40 mg/L.

The process of bacterium-toxicant-ferricyanide incubation was adapted from the one-pot assay (no toxicant removal before ferricyanide addition) by Catterall et al.^21^. In this work, 1 mL of the working inoculum of OD 1.0 was added to the sample tubes corresponding to the number of toxicant concentrations. After centrifugation at 8000*g* for 10 min, the cell pellets were redispersed in toxicants, followed by shaking incubation at 500 rpm and 37 °C for 1 h. Subsequently, 200 μL of a concentrated mixture of potassium ferricyanide (480 mM) and ammonium formate (120 mM), a substrate for respiration, was added to the cell-toxicant solution to yield the final mixture with 80 mM ferricyanide and 20 mM ammonium formate. A 15 min incubation was then allowed for ferricyanide reduction by bacterial cells. Endogenous samples were prepared in parallel without the addition of toxicants. Supernatants reporting bacterial viability were separated from the resulting solution by centrifugation at 12,000 g for 10 min and were further analyzed using the proposed measurement technique.

### Two-Electrode Platinum (Pt) Electrochemical Mini-Cell Design

The two-electrode miniaturized electrochemical cell used in this work was adapted from our group’s previous three-electrode design.^35^ As depicted in Figure 1A, it includes a Pt-foil mini-cylinder (5 mm height, 4 mm diameter), connected to both the counter-(CE) and the reference-electrode (RE) potentiostat channel. This Pt foil is positioned on the insulator cover surrounding the 3 mm Pt disk of the upward-oriented working electrode (WE). The Pt foil is placed in direct contact with the insulator part but remains out of contact with the Pt disk. This design allows the electrodes to function in a compact vessel with a capacity of 45–60 μL. The open “mouth” of the cell allows the serial measurement of samples without dismantling the cell, thus enabling continuous analysis. The proposed mini-cell configuration, moreover, offers sustainability benefits through unlimited reusability: the WE can be regularly polished, while the Pt foil CE/RE can be cleaned by easy flaming. The sample volume required was small, just tens of microliters, reducing the amount of liquid waste.

**Figure 1:**
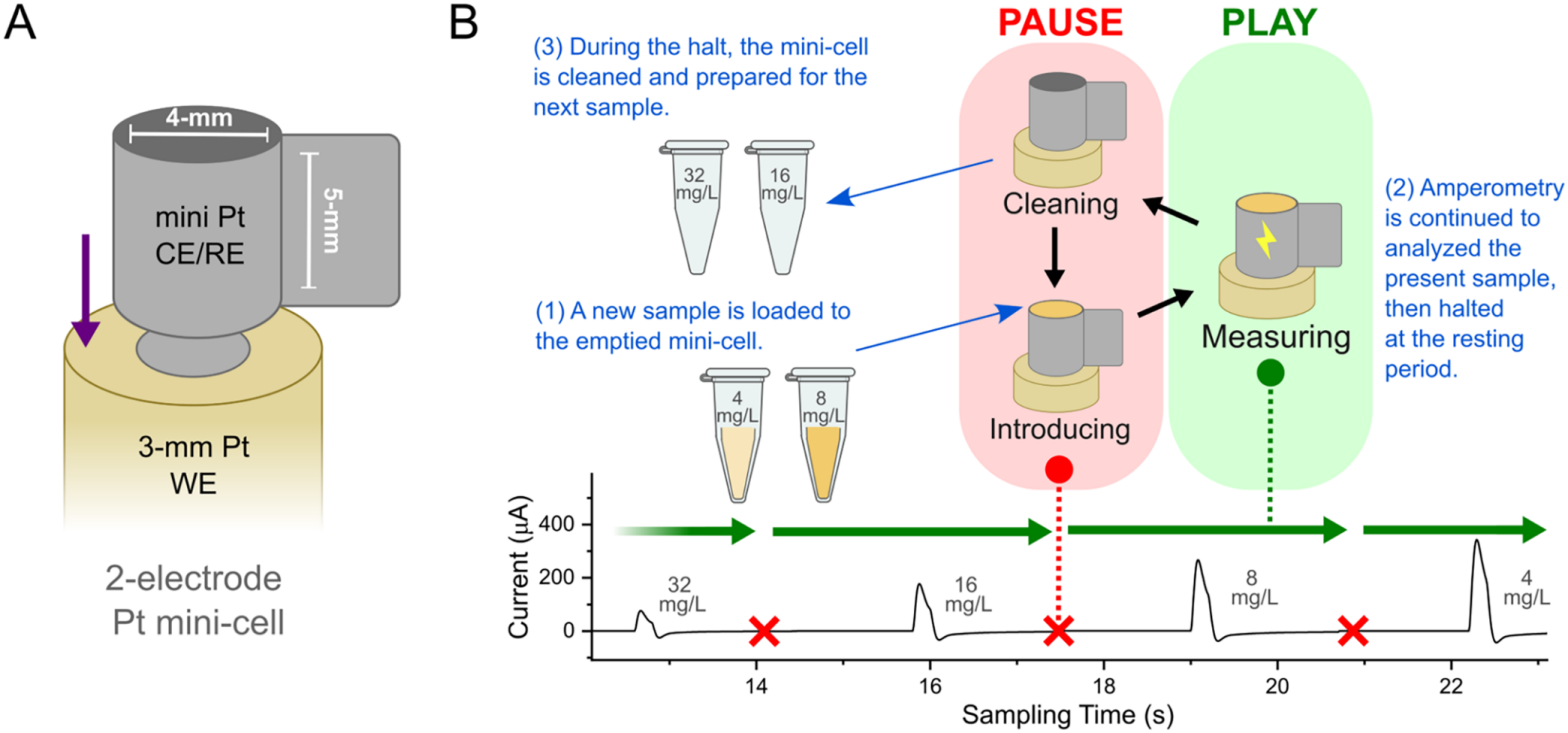
Miniaturized two-electrode Pt electrochemical cell (A) and sample-handling process for continuous-sampling amperometry in toxicity bioassays (B). The sample-handling process, termed “Pause and Play” pulse amperometry, involves: (1) Introducing a new sample during a “pause”, (2) “Play”, initiating pulse amperometry to acquire the viability response at this toxicant concentration; the amperometry is then paused again, followed by (3) Mini-cell cleaning and preparation for the next sample. Below is the model of the resulting amperogram, which displays a concentration range marked by pause periods (red crosses) and play periods (green arrows).

### Continuous Analysis

The two-electrode mini-cell enabled the sequential assessment of respiring bacterial samples with varying toxicant concentrations in a single experiment, eliminating the need for cell disassembly. This method builds upon its successful employment in previously reported electrochemical antibiotic susceptibility testing.^36^ The process involves consecutive measurements, starting from the blank, progressing through the highest to lowest toxicant concentrations, and concluding with the growth control. Pulse amperometry, consisting of a resting pulse (0.0 V, 3 s) and a detecting pulse (0.3 V, 200 ms) for each cycle, is applied in a stop-start manner, termed “Pause and Play”, based on the stages of the sampling procedure. Figure 1B illustrates the full process of the continuous measurement. The in-depth processes are described as follows: (1) as the amperometry is halted, loading of the mini-cell with 50 μL of the sample. (2) Pulse amperometry is applied from the resting pulse, followed by the detecting pulse for the sampling of ferrocyanide oxidation, and is manually paused after around 1.5 s of the resting period in the next cycle. (3) The test solution is then removed, the mini-cell thoroughly rinsed with deionized water, emptied again, and the process repeated for each toxicant concentration. Prior to each toxicity assessment set, electrode pretreatment involves ten cycles of the blank control pulse amperometry. The assessment begins with a freshly loaded blank serving as a negative control baseline, followed by a concentration-wise toxicity evaluation. The final sample in each set is the growth control, establishing the positive control baseline.

### Dose–Response Curve Fitting and IC_50_ Calculation

To calculate the %inhibition at each concentration, peak currents were extracted directly from the obtained amperograms and substituted into eq 1 below

1 $$\%\mathrm{inhibition}=1-\left(100\%\times \frac{{(i}_{n}-i_{0})}{{(i}_{G}{-i}_{0})}\right)\%$$

where *i*_*n*_ represents the peak current of a toxicant-exposed sample, *i*_0_ is that of the blank, and *i*_G_ is that of the growth control. To construct the dose–response curves, the averaged %inhibitions of three replicates were plotted against log_2_[toxicant] or [toxicant] for Pb^2+^. Lastly, nonlinear curve fitting was performed using Hill’s equation, and the IC_50_ was then extracted from the curve at the toxicant concentration corresponding to 50% inhibition.

## Results and Discussion

### The Principle of Ferricyanide-Mediated Bacterial Cell Respiration Signaling

The principle and mechanism of ferricyanide reduction by microbes have been well characterized. For *E. coli*, it has been proposed that hydrophilic ferricyanide ions ([Fe­(CN_6_)]^3–^) diffuse through outer membrane (porin) channels into the periplasmic space,^37^ where they are reduced to ferrocyanide ([Fe­(CN) _6_]^4–^) by terminal protein components of the respiratory chain.^37,38^ The ferrocyanide ions, which then diffuse extracellularly and can be electrochemically reoxidized at appropriate potentials, generate a current response. This response correlates with bacterial viability, as it reflects respiratory activity and cell population; the quantitative electrochemical response decreases proportionally when bacteria are exposed to chemicals that inhibit cell viability. Based on this principle, ferricyanide reduction in pulse amperometry has been applied in this work for detecting *E. coli* viability in the presence of toxicants (Scheme 1).

**Scheme 1:**
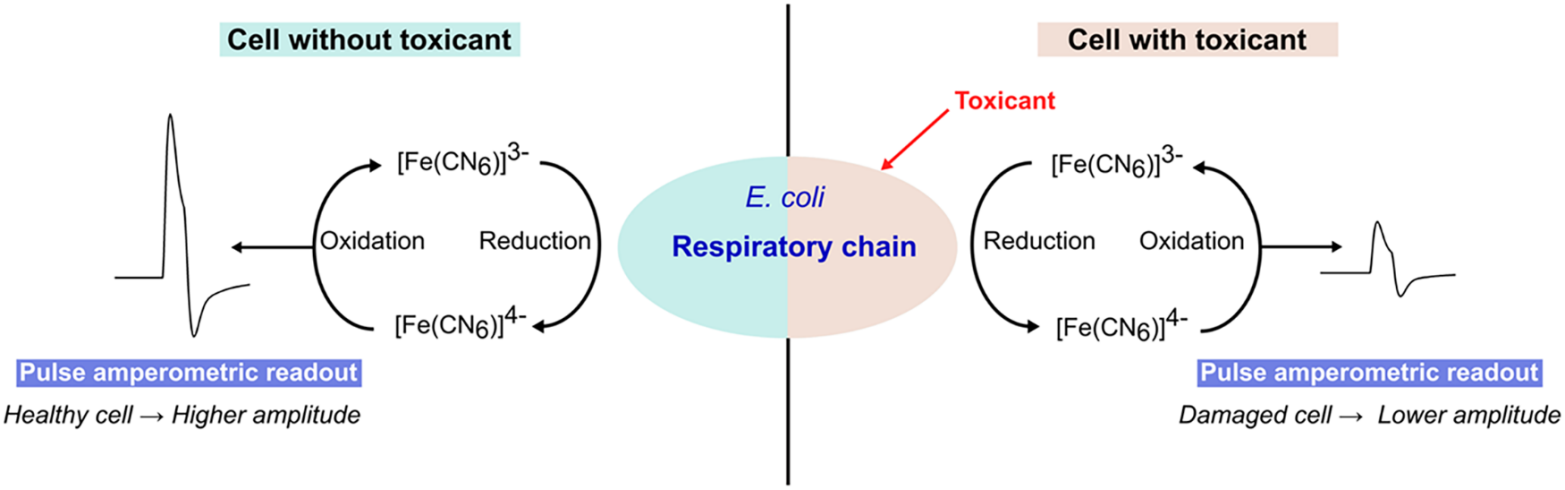
Conversion of Ferricyanide Ions [Fe­(CN_6_)]^3–^ to Ferrocyanide Ions [Fe­(CN) _6_]^4–^ by *E. coli* Respiration and the Pulse-Amperometric Readouts of the Bacterial Viability Signal with (Right) and without (Left) the Exposure to Toxicant

### Feasibility of the Two-Electrode Mini-Cell in Determining Bacterial Vitality

The fundamental analytical performance of a two-electrode mini-cell for ferricyanide/ferrrocyanide electroanalyses was described in the supplementary section of the previous work.^36^ Essentially, a well-defined duck-shaped cyclic voltammogram of the ferricyanide-ferrocyanide solution mixture was obtained with a negative potential shift of around 500 mV compared to the three-electrode mini-cell with a Ag/AgCl reference electrode. Diffusion-limited behavior, as described by the Randles-Sevcik equation, was also observed over a range of scan rates. Here, we demonstrate the capability of our method to detect the dynamic change in cell population according to the ratio of ferro/ferricyanide using pulse amperometry at 300 mV. Specifically, we tested a range of OD values from 0.2 to 1.0 with 80 mM ferricyanide, the concentration adopted in this work. This range was chosen to evaluate whether the method can detect the change in bacterial density from the lower OD to an OD of 1.0 (the baseline density in this work).

The amperogram in Figure 2A displays anodic pulses corresponding to the oxidation of ferrocyanide at each density. The amplitude of each pulse clearly correlates with the bacterial population, with higher densities producing larger oxidation pulses. As the sample density increases, a stepwise increase in the viability signal is observed. This also indicates that the decrease in bacterial respiration following toxicant exposure is detectable and confirms the feasibility of our method. A linear relationship was observed across the tested density range, with a strong correlation (*R* ^2^ = 0.9907, Figure 2B). We then proceeded to apply our method to a toxicity assessment.

**Figure 2:**
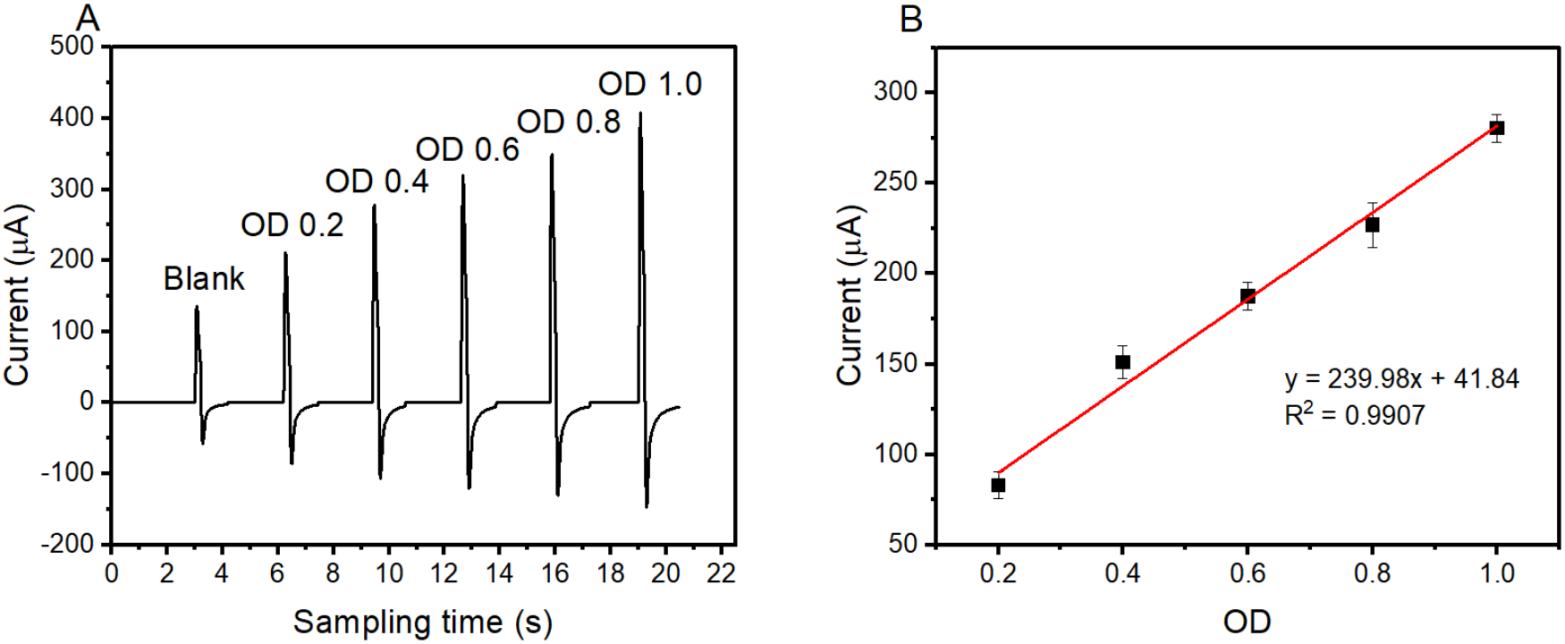
Feasibility of our method in determining the change of bacterial populations. Pulse amplitudes of ferrocyanide oxidation vary with bacterial density (A). The amperometric response is directly proportional to the density of the bacteria (B).

### Pulse-Amperometric Signal for Toxicity Assessment

Toxicity assessments were performed on incubated cell samples using the proposed continuous pulse-amperometric sampling. Overall, the toxicity characteristics of the toxicants were revealed through distinct electrochemical signals, as shown in Figures 3A,B and S1A–D. In a particular amperogram, each pulse represents an oxidation current corresponding to the rate of bacterial respiration at a specific concentration of a toxicant. Note that the horizontal axis merely indicates the sampling time from the potentiostat software and excludes the total processing time, which involves sample-handling steps. As expected, running the measurement from the samples with the expected lowest response to those with the highest response allows the raw pulse amperogram to display a rough trend of the toxicity. The pulse height variation can be observed as a pattern of the height increases from the blank (leftmost) to the samples containing the toxicant and finally to the growth control (rightmost). Importantly, the potency of each toxicant determines the height increase, resulting in an amperogram that displays the toxicity characteristic.

The instant interpretability of a toxicity trend provides experimental benefits, one of which is quick confirmation for experimentalists that the tested chemical can affect bacterial viability, thereby rapidly confirming whether it is toxic or not. Moreover, the robustness of the toxicity assay is assured at the same time if declining respiration is observed with a toxicant exposure. Another advantage is the rapid attribution of the potency of a toxicant as one can immediately determine the lowest inhibitory concentration from the amperogram; this would be especially beneficial when testing the toxicity of an unknown chemical or validating a new toxicity bioassay. If traditional amperometry were employed, this would be accessible only through data overlaying after a prolonged concentration-scanning process. Our method, on the other hand, readily provides qualitative characterization at the point of measurement due to the collective measurement, which results from simultaneous data acquisition and data compilation. Remarkably, our method employs pulsed data acquisition with a duration of 200 ms, enabling rapid reporting of toxicity characteristics in 20–30 min, even in the presence of a concentration gradient, in contrast to the traditional measurement, which can take an hour or more.

Furthermore, the resulting amperograms facilitate the calculation of the IC_50_ values. The peak current in each pulse can be extracted conveniently and substituted into eq 1 to evaluate the %inhibition relative to concentration. Though we extracted the peak current of the amperometric pulse, which contains both faradaic and nonfaradaic components, we assume that the blank subtraction (see eq 1) cancels the nonfaradaic background. This is because the sequential analyses were performed in the unchanged top-open mini-cell configuration, thereby preserving the double-layer capacitance of the electrode arrangement throughout a dose–response run. Therefore, the IC_50_ calculated from the pulse current reflects changes in the faradaic current proportional to bacterial respiration under a toxicant exposure. This highlights the practicality and power of our two-electrode mini-cell, despite its simple fabrication. Figures 3C,D and S1E–H illustrate the dose–response curves and IC_50_ of the representative toxicants.

### Toxicity Interpretations of Heavy Metal Ions and Organic Toxicants

Ni^2+^, Cd^2+^ and Pb^2+^ were selected as representative toxic metal ions and their IC_50_ values were 8.48 mg/L, 11.71 mg/L and 18.81 mg/L, respectively (Table S1), establishing the ranking as Ni^2+^ > Cd^2+^ > Pb^2+^. Note that the tested concentration range of Pb^2+^ is not 2-fold diluted and is narrower than the other toxicants (shown as a non-log scale in Figure S1F), as we found higher viability than in the growth control at lower concentrations (result not shown here). Compared to Catterall’s one-pot assay,^21^ which reported IC_50_ values of 1.9 mg/L for Ni^2+^, 7.8 mg/L for Cd^2+^, and 20.4 mg/L for Pb^2+^ (Table S1), our findings show a noticeable difference for Ni^2+^, a slight difference for Cd^2+^, and similarity for Pb^2+^. Despite these differences, the ranking remains consistent between the two studies. Furthermore, our results show a strong correlation with Ma’s one-pot assay on activated sludge containing a bacterial consortium.^31^ Ma’s reported IC_50_ values of 13.42 mg/L for Cd^2+^ and 19.8 mg/L for Pb^2+^, which are closely aligned with our findings (Table S1), indicate the robustness of our work across different bacterial systems.

Discrepancies are observed when compared to Yang’s ferricyanide-mediated two-pot assay, in which the cells were removed from the heavy metal solutions before ferricyanide incubation. Their evaluated IC_50_ values were 4.4 mg/L for Ni^2+^ and 3.7 mg/L for Cd^2+^ ^39^ (Table S1). This divergence may be due to the interaction of ferricyanide with certain heavy metals in the one-pot approach, potentially affecting the interpretation of the toxicity. Nonetheless, the one-pot method is less laborious and faster, making it a compelling choice for routine assessments.

**Figure 3:**
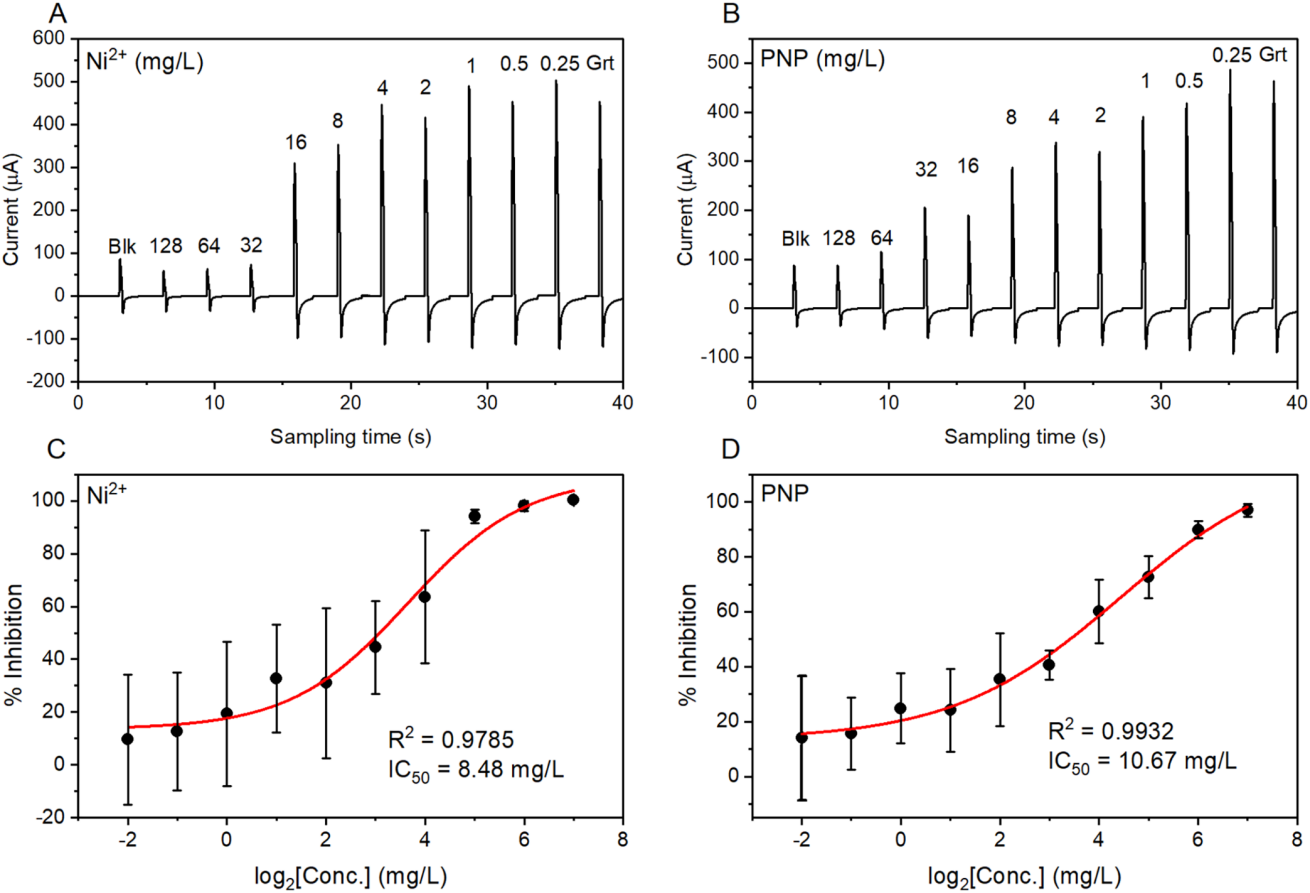
Representative dose–response amperograms for the assessment of Ni^2+^ (A) and PNP (B) toxicity. In each amperogram, the leftmost pulse represents the blank (Blk), the rightmost represents the growth control (Grt), and the pulses between represent bacterial viability responses at toxicant concentrations of 0.25 mg/L to 128 mg/L. The *X*-axis displays only the sampling time, not the overall processing time. Corresponding dose–response curves for the assessment of Ni^2+^ (C) and PNP (D) are shown with the calculated half-maximal inhibitory concentrations (IC_50_).

Evaluations of the potencies of the organic toxins were conducted. Among chlorophenols, DCP is known to be toxic to aquatic organisms^40^ and is commonly used as a model toxicant in bioassays. The IC_50_ of DCP was 4.96 mg/L (Table S1), which correlates closely with Catterall’s result of 4.88 mg/L^21^ and Ma’s evaluation of 4.889 mg/L^31^ (Table S1). However, Yong’s ferricyanide-mediated assay yielded a higher IC_50_ of 8 mg/L despite using the same *E. coli* strain^24^ (Table S1). This variation is possibly due to their use of glucose-glutamic acid as a metabolic substrate, previously shown to reduce DCP toxicity in other studies.^21,41^ The use of ammonium formate as the metabolic substrate in our work reveals its suitability with minimal interference.

The toxicities of other organic substances were likewise evaluated, namely, PNP, a toxic phenol pollutant, and carbaryl, a carbamate pesticide. The IC_50_ values were 10.67 mg/L for PNP and 35.84 mg/L for carbaryl (Table S1), establishing the toxicity ranking for organic toxicants as DCP > PNP > carbaryl. To the best of our knowledge, electrochemical toxicity bioassays for PNP and carbaryl have not been reported. Validations of PNP and carbaryl toxicity accuracy are based solely on the available Microtox half-maximal effective concentration (EC_50_), as discussed in the next section.

The comparison between our method and those of other ferricyanide-mediated electrochemical assays demonstrates a strong correlation in most cases, confirming the reliability of our system. Despite employing an unconventional procedure that differs from traditional chronoamperometric or voltammetric formats, consistent interpretations indicate that the method does not compromise accuracy. While we demonstrate the method’s feasibility for ferricyanide and *E. coli* as model redox mediator and bacterium, its adaptability to other mediators and microbes is evident from the versatility of the mediator-based approach, as reported, for instance, for yeasts^25,26^ or for a lipophilic mediator like benzoquinone,^28–30^ promising flexible applications.

### Benchmarking Against the Microtox Assay

The IC_50_ values obtained are benchmarked against the reported Microtox EC_50_ values. It is worth noting that variability in Microtox results may depend on parameters such as incubation time and the choice of test organisms. Our Ni^2+^ evaluation (8.48 mg/L) is close to Petala’s *Vibrio fischeri* result of 7.6 mg/L after 30 min of exposure and lower than their 15 min result of 17.8 mg/L^42^ (Table S1). Conversely, a significant divergence is observed compared to Codina’s 15 min assessment with *Photobacterium phosphoreum*, which reported a value of 54.67 mg/L^43^ (Table S1). For Cd^2+^, our finding (11.71 mg/L) is comparable to Petala’s 15 min result of 14.5 mg/L but about twice as high as their 30 min value of 5.3 mg/L (Table S1). Greene’s *P. phosphoreum* assay reported EC_50_ values of 25.43 mg/L and 13.79 mg/L^8^ (Table S1) for 15 min and 30 min tests, respectively, with our result aligning more closely with the 30 min value. Overall, our assessments for Ni^2+^ and Cd^2+^ exhibit good agreement with Microtox results in selected cases.

In contrast, Microtox EC_50_ values for Pb^2+^ indicate greater sensitivity compared to our findings. Tchounwou’s assay reported a value of 0.34 mg/L (15 min, *V. fischeri*)^44^, Sankaramanachi’s test yielded 1.74 mg/L (30 min, *P. phosphoreum*)^45^, and Ishaque suggested 0.455 mg/L (50 min, *V. fischeri*)^46^. All are significantly lower than our value (18.81 mg/L), regardless of the test bacteria or incubation periods (Table S1). Therefore, this discrepancy may arise from differences in the detection principles of the methods. Further studies are needed to understand the origin of this variation.

The IC_50_ values of organic toxicants in our work are likewise benchmarked against Microtox EC_50_ values. For DCP, Ricco’s assay reported 3.5 mg/L (30 min, *P. phosphoreum*)^47^, while Polo reported 6.0 mg/L (15 min, *V. fisch* *eri*)^48^ (Table S1). Despite differences in incubation times and test microorganisms, our result shows only minor variation. A similar trend is observed for PNP, where Ricco reported 8.76 mg/L, while Somasundaram reported 13.7 mg/L (5 min, *P. phosphoreum*)^49^. Our result aligns closely with these Microtox evaluations, supporting the reliability of our system.

However, a discrepancy is noticed for the carbaryl. Somasundaram reported an EC_50_ of 3.0 mg/L for a 5 min incubation,^49^ which is significantly lower than our IC_50_ value (Table S1). Although longer incubation times typically yield lower toxicity breakpoint values, their EC_50_ value is much lower than our result, albeit with a shorter incubation period (5 min vs 75 min). The variation may originate from differences in bacterial susceptibility or measurement principles. Additional approaches may be required to validate the results of our work. The toxicity of carbaryl toward different bacteria could likewise be studied to clarify the causes of the discrepancy.

The overall benchmarking suggests that our approach performs satisfactorily, as four out of six evaluations show strong alignment with Microtox. Although we propose that these discrepancies may arise from experimental factors, further research is required to identify the exact causes. Ultimately, it is worth noting that the similarities and variations in the toxicity benchmarks do not necessarily imply that one technique is inherently more accurate than the other. Instead, toxicity validation benefits from a battery of approaches.^50,51^ Different techniques can evaluate the potency of toxicants from diverse perspectives, offering a more comprehensive understanding of their toxicity characteristics. The proposed toxicity assay provides simplicity and accessibility, as it can be carried out in most electrochemistry laboratories using a straightforward, easy-to-assemble mini-cell. Its proven effectiveness and rapid access to results have been demonstrated, highlighting its value within the pool of chemical toxicity tests. Rather than replacing existing and approved regulatory assays, our methodology is ideally suited to first-line chemical toxicity screening and quick laboratory-based chemical toxicity assessments in academia and in resource-limited settings.

## Conclusion

We have introduced a continuous pulsed amperometry bioassay conducted within a miniaturized microliter-volume electrochemical cell for rapid and straightforward, yet robust, sensitive, and efficient assessments of the acute toxicity of substances. The approach was validated through toxicity evaluations of three heavy metals—Ni^2+^, Cd^2+^, and Pb^2+^—and three organic compounds: 3,5-dichlorophenol, *p*-nitrophenol, and carbaryl. Using the collected electrochemical toxicity test data, the IC_50_ values for all six toxicants were determined. A comparison of these IC_50_ results with those obtained from other ferricyanide-mediated electrochemical assays and the standard Microtox test demonstrated a good correlation, confirming the analytical reliability of our method. The proposed electrochemical toxicity bioassay combines operational simplicity with the reliability of rapid reporting of results, and the required equipment is accessible at a low cost. The methodology therefore serves as a valuable complementary tool for professionals involved in chemical risk assessments.

## Supplementary Material



## Notes

Pulse amperogram results of toxicity assessment for Cd^2+^, Pb^2+^, DCP, and carbaryl, including their fitted dose–response curves; comparison of the IC_50_ values from this work with other relevant ferricyanide-mediated electrochemical assays; and Microtox EC_50_ results (PDF)
