# Supplementary material for: A Miniaturized Two-Electrode Detection System for Convenient and Rapid Ferricyanide-Mediated Chemical Toxicity Screening
Source: Anal Chem. 2026 Mar 30;98(14):10330–7. doi: 10.1021/acs.analchem.6c00076 (PMC13084621; doi:10.1021/acs.analchem.6c00076)
Supplement: Supplementary file 1 [file ac6c00076_si_001.pdf]

# Supporting Information

## A Miniaturized Two-Electrode Detection System for Convenient and Rapid Ferricyanide-Mediated Chemical Toxicity Screening

Krittamate Buppasirakul, Wipa Suginta, and Albert Schulte*

School of Biomolecular Science and Engineering,

Vidyasirimedhi Institute of Science and Technology (VISTEC),

Wang Chan Valley, Rayong 21210, Thailand

*Corresponding author. E-mail address: albert.s@vistec.ac.th

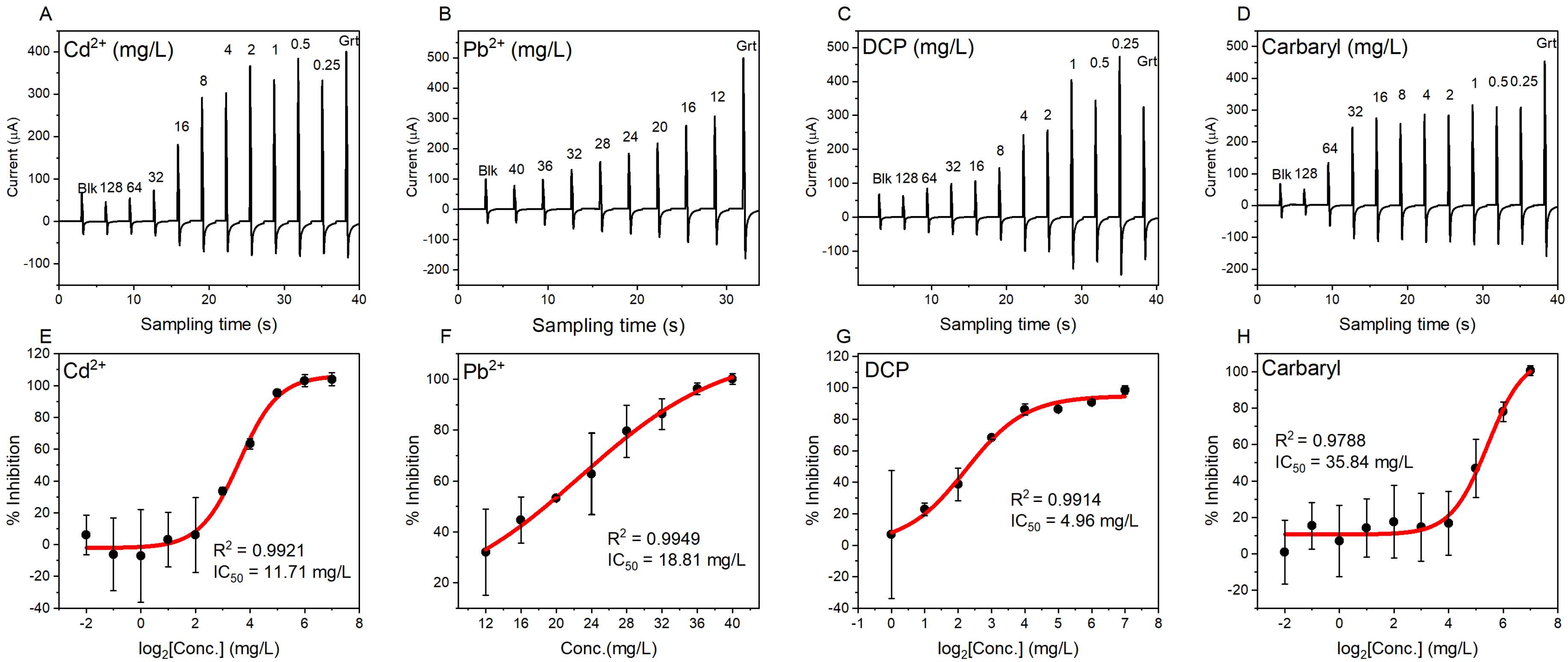


**Figure S1.** Dose-response amperograms for the toxicity assessment of $Cd^{2+}$ (A), $Pb^{2+}$ (B), DCP (C), and carbaryl (D). In each amperogram, the leftmost pulse represents the blank (Blk), the rightmost represents the growth control (Grt). The X-axis only displays the sampling time, not the overall processing time. Corresponding dose-response curves for the assessment of $Cd^{2+}$ (E), $Pb^{2+}$ (F), DCP (G), and carbaryl (H) are shown with the calculated half-maximal inhibitory concentrations ($IC_{50}$).

**Table S1.** Comparison of half-maximal inhibitory concentration ($IC_{50}$) values of $Ni^{2+}$, $Cd^{2+}$, $Pb^{2+}$, DCP, PNP, and carbaryl with the reported $IC_{50}$ values from other ferricyanide-mediated electrochemical toxicity assessments or half-maximal effective concentration ($EC_{50}$) of Microtox tests.

| Assay categories | Organism/Feature | Toxicant incubation period (min) | $IC_{50}$ or $EC_{50}$ (mg/L)* | | | | | | References |
|---|---|---|---|---|---|---|---|---|---|
| | | | $Ni^{2+}$ | $Cd^{2+}$ | $Pb^{2+}$ | DCP | PNP | Carbaryl | |
| Ferricyanide-mediated; electrochemical | *E. coli*/one-pot | 75 | 8.48 [5.67,12.01] ** | 11.71 [9.83,13.95] | 18.81 [17.3,20.32] | 4.96 [3.88,6.21] | 10.67 [8.62,13.1] | 35.84 [28.07,45.68] | This work |
| | *E. coli*/ one-pot | 75 | 1.9 | 7.8 | 20.4 | 4.88 | - | - | [1] |
| | *E. coli*/ two-pot | 60 | 4.4 | 3.7 | - | - | - | - | [2] |
| | *E. coli* | 60 | - | - | - | 8 | - | - | [3] |
| | Activated sludges | 60 | - | 13.42 | 19.8 | 4.889 | - | - | [4] |
| Microtox | *P. phosphoreum* | 15/30 | - | 25.43/13.79 | - | - | - | - | [5] |
| | | 30 | 208 | 250.9 | 1.74 | - | - | - | [6] |
| | | 15 | 54.67 | 34.25 | - | - | - | - | [7] |
| | | 5 | - | - | - | - | 13.7 | 3.0 | [8] |
| | | 30 | - | - | - | 3.5 | 8.76 | - | [9] |
| | *V. fischeri* | 15 | - | - | 0.34 | - | - | - | [10] |
| | | 15/30 | 17.8/7.6 | 14.5/5.3 | - | - | - | - | [11] |
| | | 50 | - | 0.508 | 0.455 | - | - | - | [12] |
| | | 5/15 | - | - | - | 6.8/6.0 | - | - | [13] |

* $IC_{50}$ values are reported for electrochemical toxicity bioassay while $EC_{50}$ values are reported for Microtox

** Numbers in bracket report 95% confidence interval

REFERENCES


(1) Catterall, K.; Robertson, D.; Hudson, S.; Teasdale, P. R.; Welsh, D. T.; John, R. A Sensitive, Rapid Ferricyanide-Mediated Toxicity Bioassay Developed Using Escherichia Coli. *Talanta* **2010**, *82* (2), 751–757.

(2) Yang, Y.; Fang, D.; Liu, Y.; Liu, R.; Wang, X.; Yu, Y.; Zhi, J. Problems Analysis and New Fabrication Strategies of Mediated Electrochemical Biosensors for Wastewater Toxicity Assessment. *Biosens. Bioelectron.* **2018**, *108*, 82–88.

(3) Yong, D.; Liu, C.; Yu, D.; Dong, S. A Sensitive, Rapid and Inexpensive Way to Assay Pesticide Toxicity Based on Electrochemical Biosensor. *Talanta* **2011**, *84* (1), 7–12.

(4) Ma, H.; Yong, D.; Kim, H.; Zhang, Z.; Ma, S.; Han, X. A Ferricyanide-Mediated Activated Sludge Bioassay for Determination of the Toxicity of Water. *Electroanalysis* **2016**, *28* (3), 580–587.

(5) Greene, J. C.; Miller, W. E.; Debacon, M. K.; Long, M. A.; Bartels, C. L. A Comparison of Three Microbial Assay Procedures for Measuring Toxicity of Chemical Residues. *Arch. Environ. Contam. Toxicol.* **1985**, *14* (6), 659–667.

(6) Sankaramanachi, S. K.; Qasim, S. R. Metal Toxicity Evaluation Using Bioassay and Microtox$^{TM}$. *Int. J. Environ. Stud.* **1999**, *56* (2), 187–199.

(7) Codina, J. C.; Cazorla, F. M.; Pérez-García, A.; de Vicente, A. Heavy Metal Toxicity and Genotoxicity in Water and Sewage Determined by Microbiological Methods. *Environ. Toxicol. Chem.* **2000**, *19* (6), 1552–1558.

(8) Somasundaram, L.; Coats, J. R.; Racke, K. D.; Stahr, H. M. Application of the Microtox System to Assess the Toxicity of Pesticides and Their Hydrolysis Metabolites. *Bull. Environ. Contam. Toxicol.* **1990**, *44* (2), 254–259.

(9) Ricco, G.; Tomei, M. C.; Ramadori, R.; Laera, G. Toxicity Assessment of Common Xenobiotic Compounds on Municipal Activated Sludge: Comparison between Respirometry and Microtox®. *Water Res.* **2004**, *38* (8), 2103–2110.

(10) Tchounwou  Paul B.; Reed  Lamar. Assessment of Lead Toxicity to the Marine Bacterium, Vibrio Flscheri, and to a Heterogeneous Population of Microorganisms Derived from the Pearl River in Jackson, Mississippi, USA. **1999**, *14* (2), 51–62.

(11) Petala, M.; Tsiridis, V.; Kyriazis, S.; Samaras, P.; Kungolos, A.; Sakellaropoulos, G. Evaluation of Toxic Response of Heavy Metals and Organic Pollutants Using Microtox Acute Toxicity Test. *Proc. 9th Int. Conf. Environ. Sci. Technol.* **2005**.

(12) Ishaque, A. B.; Johnson, L.; Gerald, T.; Boucaud, D.; Okoh, J.; Tchounwou, P. B. Assessment of Individual and Combined Toxicities of Four Non-Essential Metals (As, Cd, Hg and Pb) in the Microtox Assay. *International Journal of Environmental Research and Public Health*. 2006, pp 118–120.

(13) Polo, A. M.; Tobajas, M.; Sanchis, S.; Mohedano, A. F.; Rodríguez, J. J. Comparison of Experimental Methods for Determination of Toxicity and Biodegradability of Xenobiotic Compounds. *Biodegradation* **2011**, *22* (4), 751–761.
